# *Lippia grata* Schauer: Essential Oil and Phytoceutical Thymol Antioxidants and Neuroprotectors with Inhibition of Acetylcholinesterase and Depressive Behaviors in Adult Zebrafish (*D. rerio*)

**DOI:** 10.1021/acschemneuro.5c00331

**Published:** 2025-09-30

**Authors:** Luiz F. Wemmenson G. Moura, Maria Rayane C. de Oliveira, Gabriela A. do Nascimento, João Gabriel L. da Silva, Paulo A. T. Coelho, Lorena S. Lima, Sacha Aubrey A. R. Santos, Keciany A. de Oliveira, Solange de O. Pinheiro, Francisco Lucas A. Batista, Hamilton M. Ishiki, Antonio E. Vieira-Neto, Erlândia A. M. Queiroz, Stenio F. Félix, Wildson Max B. da Silva, Lucas S. Frota, Sara Ingrid C. G. Barbosa, Selene M. de Morais, Maria Izabel F. Guedes, Henrique D. M. Coutinho, Renalison Farias-Pereira, Cassia M. M. da Silva, Ramon da S. Raposo, Adriana R. Campos, Francisco Ernani A. Magalhães

**Affiliations:** † State University of Ceara, Department of Nutrition and Health, Itaperi Campus, CEP 60714-903 Fortaleza-CE, Brazil; †† State University of Ceara, Graduate Program of Nutrition and Health, Itaperi Campus, CEP 60714-903 Fortaleza-CE, Brazil; § State University of Ceara, Graduate Program of Physiological Sciences, Itaperi Campus, CEP 60714-903 Fortaleza-CE, Brazil; §§ State University of Ceara, Biotechnology and Molecular Biology Laboratory (LBBM), CEP 60714-903 Fortaleza, Brazil; ∥ State University of Ceara, Inorganic Chemistry Laboratory, Itaperi Campus, CEP 60714-903 Fortaleza-CE, Brazil; ⊥ State University of Ceara, Laboratory of Bioprospecting of Natural Products and Biotechnology, CECITEC Campus, CEP 60.660-000 Tauá-CE, Brazil; ‡ University of Fortaleza, Center for Experimental Biology, CEP 60.811-650 Fortaleza, Ceará, Brazil; ## State University of Ceara, Faculty of Sciences of the Central Sertão (FACISC), FACISC Campus, CEP 63800-000 Quixeramobim, Ceará, Brazil; # Federal Institute of Ceara, Chemical Research Group of Natural Products and Biological Activities of the Semiarid Region, Iguatu Campus, CEP 63.500-000 Iguatu, Ceará, Brazil; ∇ Acaraú Valley State University, Department Chemistry, Sobral Campus, CEP 62.040-370 Sobral, Ceará, Brazil; ○ State University of Ceara, Graduate Program of Natural Sciences, Itaperi Campus, CEP 60714-903 Fortaleza, Brazil; ◆ State University of Ceara, Northeast Biotechnology Network (RENORBIO), Chromatographic and Spectroscopic Analysis Laboratory, CEP 60714-903 Fortaleza, Ceará, Brazil; ¶ Department of Biological Sciences, 28128 Kean University, Kean Union Campus, Union, New Jersey 07083, United States; △ Regional University of Cariri, Graduate Program of Biological Chemistry, Microbiology and Molecular Biology Laboratory, CEP 63105-000 Crato, Ceará, Brazil; ▲ Estácio de Sá University, Jóquei Clube Campus, CEP 60.510-111 Fortaleza, Ceará, Brazil; ▲▲ Graduate Program in Biotechnology of Federal University of Ceará, Sobral Campus, CEP 60.510-111 Fortaleza, Ceará, Brazil

**Keywords:** Natural Products, Essential Oil, Thymol, Depression, adult zebrafish (D. rerio)

## Abstract

Depression, a growing mental disorder, affects millions of people globally and faces treatment challenges due to the low efficacy and adverse effects of conventional antidepressants. In this context, medicinal plants such as *Lippia grata* Schauer, endemic to Brazil and recognized for their therapeutic properties, stand out as promising alternatives for developing more effective and safe treatments. Therefore, this work reports the standardization of the depression model in adult zebrafish (aZF), in addition to evaluating the antidepressant effect of *Lippia grata* essential oil (EOLg) and the phytoceutical thymol, as well as their potential neuromodulatory mechanisms and *in vitro* antioxidant and anticholinesterase (AChE) activities. Initially, aZF were treated with fluoxetine (Flx) or EOLg or thymol or vehicle and subjected to Toxicity and Open Field tests. After 1 h of the same treatments, in other aZF groups, the animals were individually immersed in EtOH for 30 min, with the exception of the naïve group. Subsequently, the aZF were subjected to the Zebrafish Tail Immobilization Test, and the antidepressant effect was characterized by an increase in Mobility Time (s), MT. The possible mechanisms of action were investigated through the administration of antagonists of the serotonergic system. The antioxidant capacity and acetylcholinesterase (AChE) inhibitory effect were assessed *in vitro*, including the determination of IC_50_ values for the DPPH and ABTS radicals and the AChE enzyme. Furthermore, molecular docking simulations of thymol with 5-HT receptors were investigated. The toxicological results indicated that the samples are safe against aZF. Flx presented an antidepressant effect, but with a sedative effect, while EOLg and thymol exhibited an antidepressant effect, without a sedative effect and via serotonergic systems. *In vitro* tests showed antioxidant and neuroprotective potential against AChE in the samples analyzed. Furthermore, *in silico* tests confirmed the affinity of thymol for the 5-HT_1B_, 5-HT_2A_, 5-HT_2C_, and 5-HT_3A_ receptors. These findings reinforce the importance of *Lippia Grata* essential oil as a source of the phytoceutical thymol with neuroprotective potential in neurological disorders.

## Graphical abstract

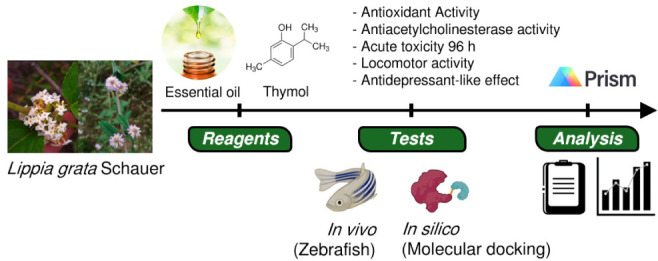

## 1 Introduction

Depression is a prevalent psychiatric condition marked by enduring emotional distress, diminished interest in routine or pleasurable activities, and impairments in cognitive function. According to data from the World Health Organization, its prevalence has increased significantly globally, affecting approximately 28 million people each year.^1^ It is the third most costly disease worldwide.^2^

From a clinical perspective, depression presents with symptoms including chronic pessimism, reduced ability to experience pleasure (anhedonia), appetite suppression, sleep irregularities, and impaired concentration. Notably, individuals with major depressive disorder face a heightened risk of self-injurious behavior and suicide, posing a serious concern for public health and societal well-being.^3^

Currently, nearly one-third of individuals with depression show limited or no response to conventional antidepressant treatments.^4^ Serotonergic drugs are known to produce persistent neuroplastic modifications in brain regions such as the hippocampus and prefrontal cortex (PFC), which are involved in processes like associative learning and the extinction of fear responses in chronic stress or trauma-related conditions.^5^

Importantly, side effects like dizziness, headaches, cognitive slowing, episodes of fainting, sexual dysfunction, and gastrointestinal symptoms such as nausea and vomiting frequently contribute to the premature discontinuation of treatment, even among patients who initially benefit from antidepressant use. Thus, identifying new therapeutic targets and developing more effective antidepressants are of great relevance and urgency.^6^

In this context, plant resources have played an essential role throughout human history. After meeting basic needs such as food and shelter, humans began to explore the medicinal properties of plants, using them as fundamental tools in treating various diseases.^7^ Among these plants, *Lippia grata* Schauer belongs to the Verbenaceae family.^8^ *Lippia grata* is a medicinal species native to Brazil, commonly found in biomes such as the Caatinga, Rupestrian Fields, and Cerrado. Traditionally, it has been employed in folk medicine for the treatment of infectious diseases, attributed to its antimicrobial, antiseptic, and wound-healing properties.^9^ Its essential oil (EO), obtained from the leaves, is notably rich in monoterpenes and sesquiterpenes. The main compounds identified in this essential oil include thymol, carvacrol, alpha-pinene, and p-cymene.^10^

In recent years, essential oils have emerged as promising candidates in the search for novel antidepressant agents. Their primary constituents are volatile aromatic compounds that can swiftly affect emotional and behavioral responses through direct activation of the olfactory pathways. Moreover, these molecules possess the ability to cross the blood-brain barrier, modulating the synthesis and release of neurotransmitters and hormones involved in depressive states.^11^

The zebrafish (*Danio rerio*) has emerged as an experimental model species widely used in several research areas, including environmental studies, basic biology, genetics, behavior, neuroscience, toxicology, and translational research. Its versatility and relevance have made it a valuable tool for scientific investigations across multiple fields.^12^

Based on the above, this study aimed to standardize the depression model in adult zebrafish (aZF) and evaluate the antidepressant effects of the essential oil from *Lippia grata* leaves (EOLg) and the phytoceutical thymol, along with potential neuromodulation mechanisms, as well as its antioxidant and anticholinesterase (AChE) activity *in vitro*.

## 2 Results

### 2.1 Nonclinical Safety Assessment

#### 2.1.1 Acute Toxicity—96 h

EOLg and thymol were administered per os (*p.o.*) at concentrations of 0.01 or 0.1 or 1.0 mg/mL in a volume of 20 μL, while fluoxetine was given per os (*p.o.*) at concentrations of 5.0 or 10 or 15 mg/mL, also in 20 μL volumes, did not demonstrate toxicity in relation to aZF during the analyses conducted over 96 h, as determined by the Spearman-Karber calculation method. The findings demonstrate that the lethal concentration (LC_50_) for both compounds was superior to 1.0 and 15 mg/mL, respectively (Table 1).

**Table 1:** Results of Acute Toxicity Tests of the Test Samples against Adult Zebrafish

	Adult Zebrafish Mortalities				
Sample	Vehicle	C1^b^	C2^c^	C3[Table-fn tbl1fn4]	96 h of Analysis LC_50_ (mg/mL)/IV
EOLg	0	0	0	0	>1.0
Thymol	0	0	0	0	>1.0
Flx^**a‑c**^	0	0	0	0	>15

LgEO—*Lippia grata* essential oil (EOLg). Flx—fluoxetine. Vehicle—3% DMSO (Control; 20 μL; *p.o.*). C1—0.01 mg/mL; 20 μL; *p.o.*

a 5.0 mg/mL; 20 μL; *p.o.*; C2—0.1 mg/mL; 20 μL; *p.o.*

b 10 mg/mL; 20 μL; *p.o.*; C3—1.0 mg/mL; 20 μL; *p.o.*

c 15 mg/mL; 20 μL; administered per os (*p.o.*); LC50—lethal concentration required to kill 50% of adult zebrafish; IV—confidence interval.

#### 2.1.2 Locomotor Activity (Open Field Test)

Our research has revealed that EOLg, at various concentrations, did not induce a sedative effect. This is a significant finding, as it provides valuable insights into the pharmacological properties of EOLg. Animals administered with EOLg per os (*p.o.*) at concentrations of 0.01 or 0.1 or 1.0 mg/mL in a volume of 20 μL did not exhibit significant changes in the locomotor activity (LA) of adult zebrafish (aZF) [(*q* = 15.79; *q* = 15.12; *q* = 15.16) *p* < 0.0001 *vs.* DZP], which was different from the effect observed in animals treated per os (*p.o.*) with diazepam (DZP) at a concentration of 10 mg/mL and a volume of 20 μL, the sedative control (*q* = 17.82, *p* < 0.0001 *vs.* Naive; *q* = 15.30, *p* < 0.0001 *vs.* Vehicle), as shown in Figure 1A (F_5, 30_ = 42.84).

**Figure 1:**
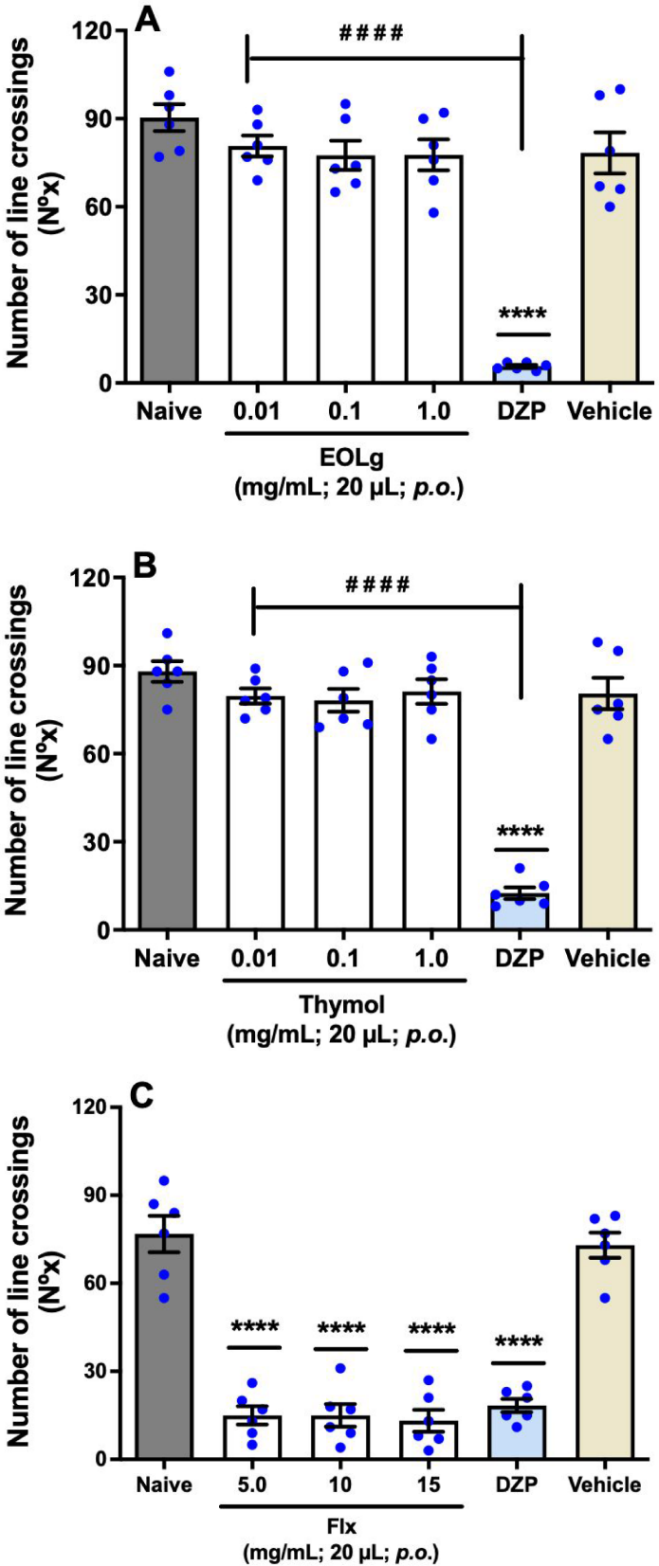
Effect of essential oil from *Lippia grata* leaves, EOLg (A), thymol (B) and fluoxetine, Flx (C) on locomotor activity of adult zebrafish (*Danio rerio*) during the Open Field Test (0–5 min). Naive: untreated animals; Vehicle: 3% DMSO. Data are presented as mean ± standard error of the mean (S.E.M.) for six animals per group. Statistical analysis was performed using ANOVA followed by Tukey’s post hoc test (****p < 0.001 *vs.* Naive or Vehicle; ^**# # # #**^ *p* < 0.001 *vs.* DZP).

Thymol also did not exhibit a sedative effect, as animals treated with thymol (0.01 or 0.1 or 1.0 mg/mL; 20 μL; *p.o.*) did not show significant alterations in the locomotor activity (LA) of aZF [(*q* = 17.99; *q* = 17.59; *q* = 18.39) *p* < 0.0001 *vs.* DZP], which was different from the effect observed in animals treated with diazepam (DZP; 10 mg/mL; 20 μL; *p.o.*), the sedative control (*q* = 20.22, *p* < 0.0001 *vs.* Naive; *q* = 18.21, *p* < 0.0001 *vs.* Vehicle), as shown in Figure 1B (F_5, 30_ = 57.73). Flx exhibited a sedative effect in aZF, as Flx was administered per os (*p.o.*) at concentrations of 5.0 or 10 or 15 mg/mL in a volume of 20 μL, resulting in a significant decrease in locomotor activity (LA) of aZF [(*q* = 0.8164; *q* = 0.8164; *q* = 1.265) *p* > 0.05 *vs.* DZP], similar to the effect observed in animals treated per os (*p.o.*) with diazepam (DZP) at a concentration of 10 mg/mL and a volume of 20 μL in animals treated per os (*p.o.*) with diazepam (DZP) at a concentration of 10 mg/mL and a volume of 20 μL, the sedative control (*q* = 14.33, *p* < 0.0001 *vs.* Naive; *q* = 13.39, *p* < 0.0001 *vs.* Vehicle), as shown in Figure 1C (F_5, 30_ = 56.96).

### 2.2 Antioxidant and Antiacetylcholinesterase Activity

The results obtained indicate that the samples exhibited a significant radical inhibition capacity for both the ABTS radical and the DPPH radical, with the most notable being EOLg 1.0 mg/mL with mean inhibitory concentration (IC_50_) values of 8.37 ± 0.57 μg.mL^–1^. For the inhibition of acetylcholinesterase, all samples also showed high inhibition (IC_50_ < 20 μg.mL^–1^) of the enzyme, with the most notable being EOLg 1.0 mg/mL with IC_50_ of 11.34 ± 0.87 μg.mL^–1^ (Table 2).

**Table 2:** *In vitro* Antioxidant and Anticholinesterase (AChE) Effect of EOLg and Thymol

Samples	IC_50_ DPPH^•^ (μg.mL^–1^)	IC_50_ ABTS^+•^ (μg.mL^–1^)	IC_50_ AChE (μg.mL^–1^)
Quercetin (Standard)	2.74 ± 0.08	3.98 ± 0.13	5.48 ± 0.03
Gallic Acid (Standard)	1.94 ± 0.27	13.01 ± 0.03	-
Galantamine (Standard)	-	-	5.82 ± 0.02
Physostigmine (Standard)	-	-	6.68 ± 0.08
Thymol 0.01 mg/mL	15.38 ± 0.43	36.55 ± 0.18	25.54 ± 0.18
Thymol 0.1 mg/mL	14.89 ± 0.22	18.44 ± 0.23	19.64 ± 0.29
Thymol 1.0 mg/mL	11.89 ± 0.21	19.47 ± 0.15	16.81 ± 0.37
EOLg 0.01 mg/mL	10.86 ± 0.25	17.08 ± 0.30	19.54 ± 0.41
EOLg 0.1 mg/mL	10.04 ± 0.71	15.31 ± 0.17	15.14 ± 0.32
EOLg 1.0 mg/mL	8.37 ± 0.57	12.12 ± 0.32	11.34 ± 0.87
IC_50_ - mean inhibitory concentration.			

### 2.3 Antidepressant-Like Effect

Flx mitigated the depressive effects induced by 1% EtOH in adult zebrafish (aZF), as animals treated per os (*p.o.*) with Flx at concentrations of 5.0 or 10 or 15 mg/mL in a volume of 20 μL showed increased the MT of aZF in the ZTI (5 min), significantly [(*q* = 14.18; *q* = 12.58; *q* = 10.30) *p* < 0.00001 *vs.* Vehicle] different from the MT of animals treated with the Vehicle (3% DMSO). The MT of animals treated with Flx (5.0 or 10 or 15 mg/mL; 20 μL; *p.o.*) was significantly [(*q* = 0.2586; *q* = 1.857; *q* = 4.138) *p* < 0.05 *vs.* Naive] similar to the MT of untreated animals (Naive). Therefore, the lowest effective dose of Flx used as antidepressant control was 5.0 mg/mL, Figure 2A (F_4, 25_ = 25.40).

**Figure 2:**
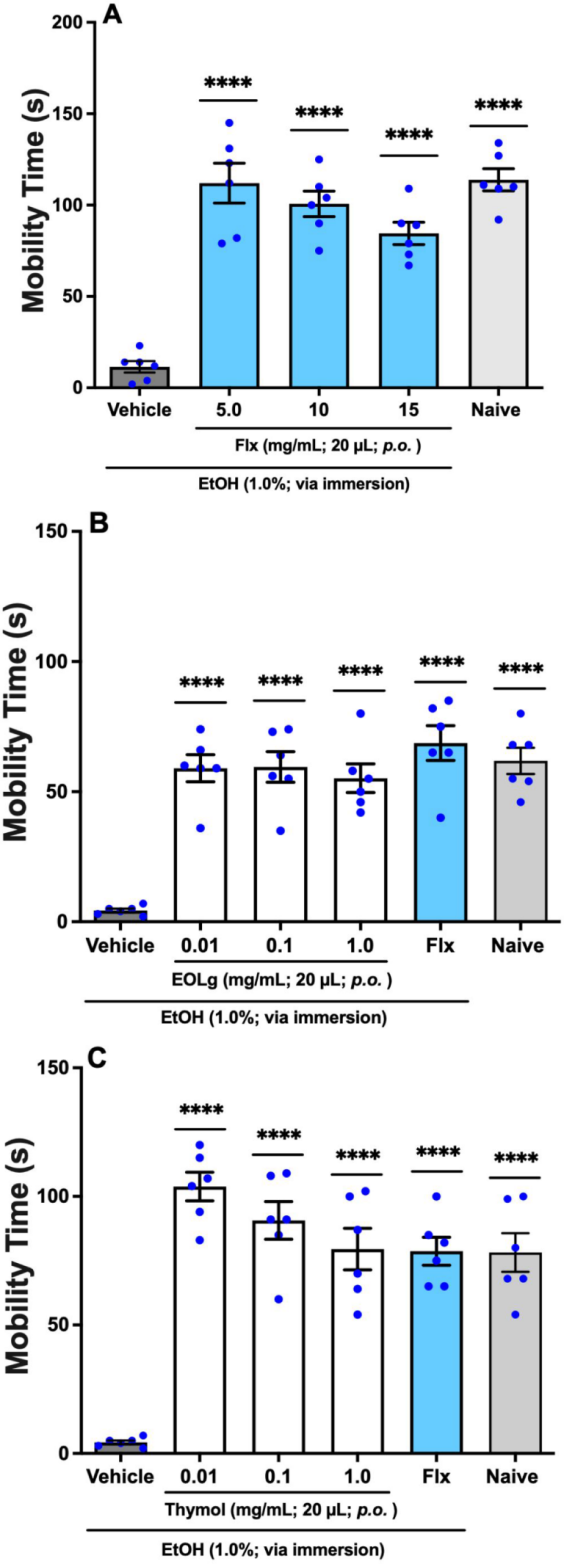
Antidepressant-like effect of (A) fluoxetine (Flx), (B) EOLg, and (C) thymol in the Zebrafish Tail Immobilization Test (ZTI) during 5 min of analysis. Vehicle: control group receiving 3% DMSO (20 μL, per os, *p.o.*). Naive: untreated animals. Data are expressed as mean ± standard error of the mean (S.E.M.) for six animals per group. Statistical analysis was performed using ANOVA followed by Tukey’s post hoc test (****p < 0.0001 *vs.* Vehicle).

Regarding EOLg, it demonstrated an antidepressant-like activity in adult zebrafish (aZF), as animals administered per os (*p.o.*) with EOLg at concentrations of 0.01 or 0.1 or 1.0 mg/mL in a volume of 20 μL showed increased the MT of animals in the ZTI (5 min), significantly [(*q* = 10.51; *q* = 10.60; *q* = 9.771) *p* < 0.0001 *vs.* Vehicle] different from the MT of animals treated with the Vehicle (3% DMSO) and significantly [(*q* = 0.5446; *q* = 0.4485; *q* = 1.286) *p* < 0.05 *vs.* Naive] similar to the MT of untreated animals (Naive). Such antidepressant-like effect of EOLg was significantly [(*q* = 1.858; *q* = 1.762; *q* = 2.595) *p* < 0.05 *vs.* Flx] similar to the antidepressant effect observed with Flx administered per os (*p.o.*) at 5.0 mg/mL in a volume of 20 μL, used as antidepressant control (*q* = 12.37, *p* < 0.0001 *vs.* Vehicle; *q* = 1.313, *p* < 0.05 *vs.* Naive), Figure 2B (F_5, 30_ = 20.39).

Thymol similarly exhibited an antidepressant-like activity in adult zebrafish (aZF), as animals administered per os (*p.o.*) with thymol at concentrations of 0.01 or 0.1 or 1.0 mg/mL in a volume of 20 μL showed an increase in MT of animals in the ZTI (5 min), significantly [(*q* = 15.83; *q* = 13.73; *q* = 11.96) *p* < 0.0001 *vs.* Vehicle] different from the MT of animals treated with the Vehicle (3% DMSO) and significantly [(*q* = 4.083; *q* = 1.988; *q* = 0.2121) *p* < 0.05 *vs.* Naive] similar to the MT of untreated animals (Naive). Such antidepressant-like effect of EOLg was significantly [(*q* = 4.003; *q* = 1.909; *q* = 0.1326) *p* < 0.05 *vs.* Flx) similar to the antidepressant effect of Flx (5.0 mg/mL; 20 μL; *p.o.*), used as antidepressant control (*q* = 11.82, *p* < 0.0001; *vs.* Vehicle; *q* = 0.07954, *p* < 0.05 *vs.* Naive), Figure 2C (F_5, 30_ = 30.76).

#### 2.3.1 Neuromodulation via the 5-HT_2A_

The antidepressant-like activity of EOLg, administered per os (*p.o.*) at a concentration of 0.01 mg/mL in a 20 μL volume, was significantly reversed (*q* = 8.370, *p* < 0.0001; EOLg *vs.* Cypro + EOLg) by cyproheptadine (Cypro) at 0.8 mg/mL, orally administered in 20 μL an antagonist of the 5-HT_2A_ system.^13^ This effect was also significantly comparable (*q* = 0.7300, *p* < 0.05; Cypro + EOLg *vs.* Cypro + Flx) to that of fluoxetine (Flx; antidepressant control; 5.0 mg/mL; 20 μL; *p.o.*). However, fluoxetine’s effect was notably reversed (*q* = 13.77, *p* < 0.0001; Flx *vs.* Cypro + Flx) by cyproheptadine, as shown in Figure 3 (F _6, 35_ = 39.01). These findings indicate that the reversal of EOLg’s antidepressant-like effect following cyproheptadine pretreatment implicates involvement of the serotonergic 5-HT_2A_ receptor in its mechanism of action.

**Figure 3:**
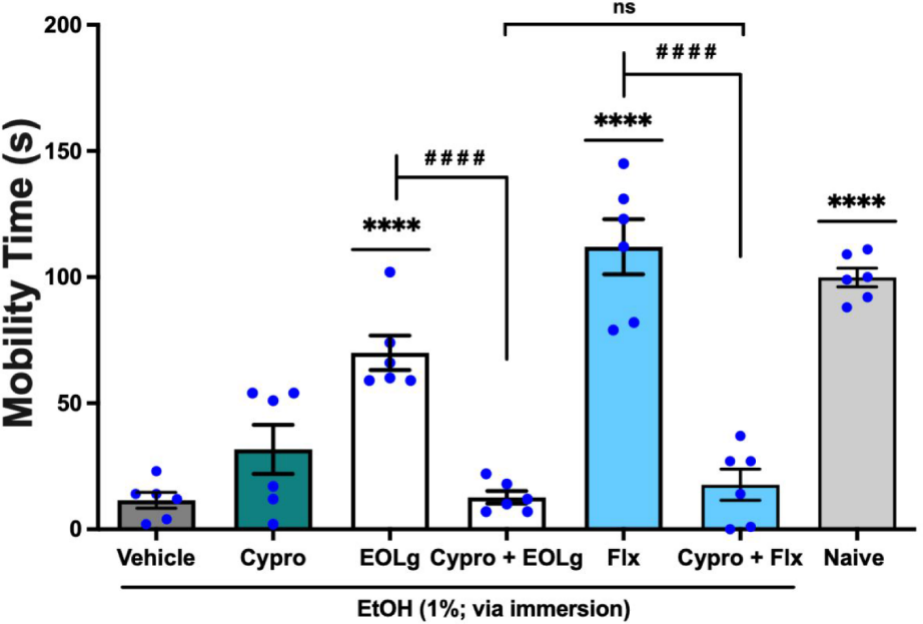
Effect of cyproheptadine (Cypro; 5-HT_2A_ receptor antagonist; 0.8 mg/mL; 20 μL; *p.o.*) on the antidepressant-like activity of essential oil from *Lippia grata* leaves (EOLg; 20 μL; *p.o.*) in the Zebrafish Tail Immobilization Test (ZTI) during the 0–5 min analysis period. Naive: untreated animals. Flx: fluoxetine, used as the reference antidepressant (5.0 mg/mL; 20 μL; *p.o.*). Vehicle: 3% DMSO solution administered orally (20 μL). Data are expressed as mean ± standard error of the mean (S.E.M.) for six animals per group. Statistical analysis was performed using ANOVA followed by Tukey’s post hoc test (****p < 0.001 *vs.* Vehicle; ^**# # # #**^ *p* < 0.0001 *vs.* EOLg or Flx; ns—not significant = *p* > 0.05).

The antidepressant-like activity of thymol, administered per os (*p.o.*) at 0.01 mg/mL in a 20 μL volume was also significantly reversed (*q* = 13.51, *p* < 0.0001; thymol *vs.* Cypro + thymol) by cyproheptadine (Cypro) at 0.8 mg/mL, orally administered in 20 μL an antagonist of the 5-HT_2A_ system.^13^ This effect was also significantly (*q* = 1.104, *p* < 0.05; Cypro + thymol *vs.* Cypro + Flx) similar to the effect of Fluoxetine (Flx), used as a standard antidepressant, administered per os at 5.0 mg/mL in a volume of 20 μL, which was also significantly reversed (*q* = 13.58, *p* < 0.0001; Flx *vs.* Cypro + Flx) by cyproheptadine, Figure 4 (F _6, 35_ = 48.10). Thus, the reversal of the antidepressant-like effect of Thymol by pretreatment with cyproheptadine suggests that its effects depend on the serotonergic 5-HT_2A_ receptor.

**Figure 4:**
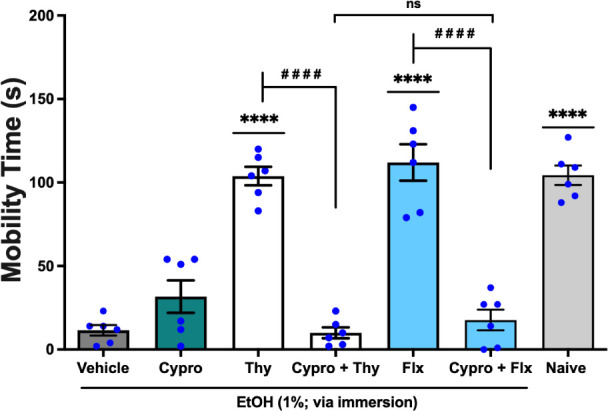
Effect of cyproheptadine (Cypro; 5-HT_2A_ receptor antagonist; 0.8 mg/mL; 20 μL; *p.o.*) on the antidepressant-like activity of thymol (Thy; 0.01 mg/mL; 20 μL; p.o.) in the Zebrafish Tail Immobilization Test (ZTI), evaluated over a 5 min period (0–5 min). Naive: untreated animals. Flx: fluoxetine, employed as the reference antidepressant (5.0 mg/mL; 20 μL; *p.o.*). Vehicle: 3% DMSO, orally administered in a volume of 20 μL. Data are expressed as mean ± standard error of the mean (S.E.M.) for six animals per group. Statistical analysis was performed using ANOVA followed by Tukey’s post hoc test (****p < 0.001 *vs.* Vehicle; ^**# # # #**^ *p* < 0.0001 *vs.* thymol or Flx; ns – not significant = *p* > 0.05).

#### 2.3.2 Neuromodulation via the 5-HT_1_ and 5-HT_2*A*/2C_

The antidepressant-like activity of EOLg, administered per os (*p.o.*) at 0.01 mg/mL in a 20 μL volume was significantly reversed (*q* = 8.417, *p* < 0.0001; EOLg *vs.* Piz + EOLg) by pizotifen (Piz) at 0.8 mg/mL, orally administered in 20 μL an antagonist of the serotonergic system 5-HT_1_ and 5-HT_2*A*/2C_.^13^ This effect was significantly (*q* = 1.853, *p* < 0.05; Piz + EOLg *vs.* Piz + Flx) similar to the effect of Fluoxetine (Flx), used as a standard antidepressant, administered per os at 5.0 mg/mL in a volume of 20 μL, which was also significantly reversed (*q* = 16.94, *p* < 0.0001; Flx *vs.* Piz + Flx) by pizotifen, Figure 5 (F _6, 35_ = 51.68). Thus, the reversal of the antidepressant-like effect of EOLg by pretreatment with pizotifen suggests that its effects depend on the 5-HT_1_ and 5-HT_2*A*/2C_ serotonin receptors.

**Figure 5:**
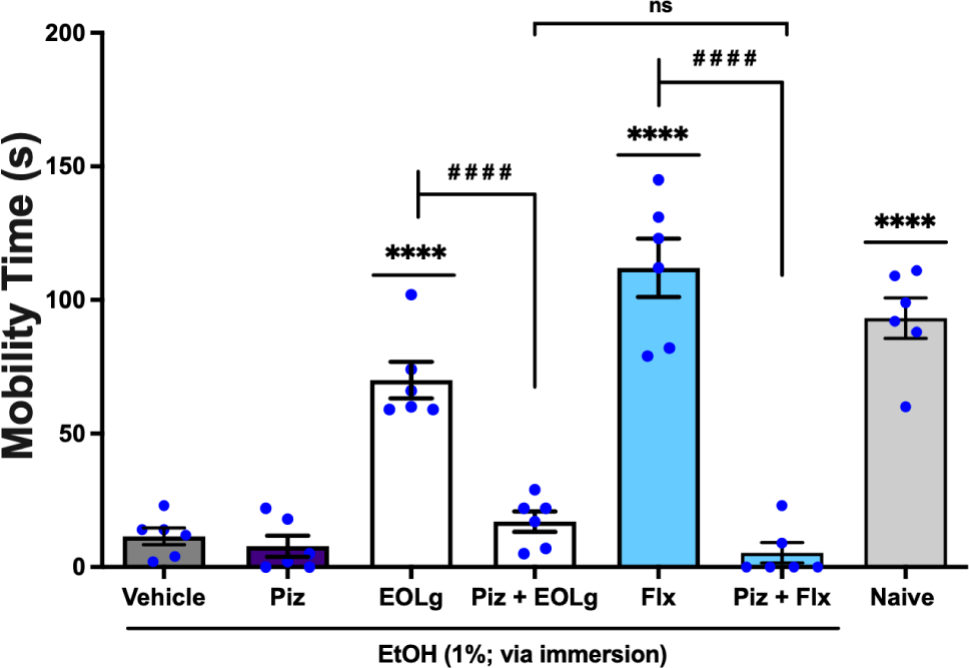
Effect of pizotifen (Piz; antagonist of 5-HT_1_ and 5-HT_2A/2C_ receptors; 0.8 mg/mL; 20 μL; *p.o.*) on the antidepressant-like activity of essential oil from *Lippia grata* leaves (EOLg; 0.01 mg/mL; 20 μL; *p.o.*) in the Zebrafish Tail Immobilization Test (ZTI) over a 0–5 min period. Naïve: untreated animals. Flx: fluoxetine, employed as the reference antidepressant (5.0 mg/mL; 20 μL; *p.o.*). Vehicle: 3% DMSO, orally administered in a volume of 20 μL. Data are presented as mean ± standard error of the mean (S.E.M.) for six animals per group. Statistical analysis was conducted using ANOVA followed by Tukey’s post hoc test (****p < 0.0001 *vs.* Vehicle; ^**# # # #**^ *p* < 0.0001 *vs.* EOLg or Flx; ns – not significant = *p* > 0.05).

The antidepressant-like activity of thymol was observed following per os administration at 0.01 mg/mL in 20 μL was significantly reversed (*q* = 14.97, *p* < 0.0001; Thy *vs.* Piz + Thy) by pizotifen (Piz) at 0.8 mg/mL, orally administered in 20 μL an antagonist of the serotonergic system 5-HT_1_ and 5-HT_2*A*/2C_.^13^ This effect was significantly (*q* = 1.692, *p* < 0.05; Piz + thymol *vs.* Piz + Flx) similar to the effect of Fluoxetine (Flx), used as a standard antidepressant, administered per os at 5.0 mg/mL in a volume of 20 μL, which was also significantly reversed (*q* = 18.05, *p* < 0.0001; Flx *vs.* Piz + Flx) by pizotifen, Figure 6 (F _6, 35_ = 76.98). Thus, the reversal of the antidepressant-like effect of EOLg by pretreatment with pizotifen suggests that its effects depend on the 5-HT_1_ and 5-HT_2*A*/2C_ serotonin receptors.

**Figure 6:**
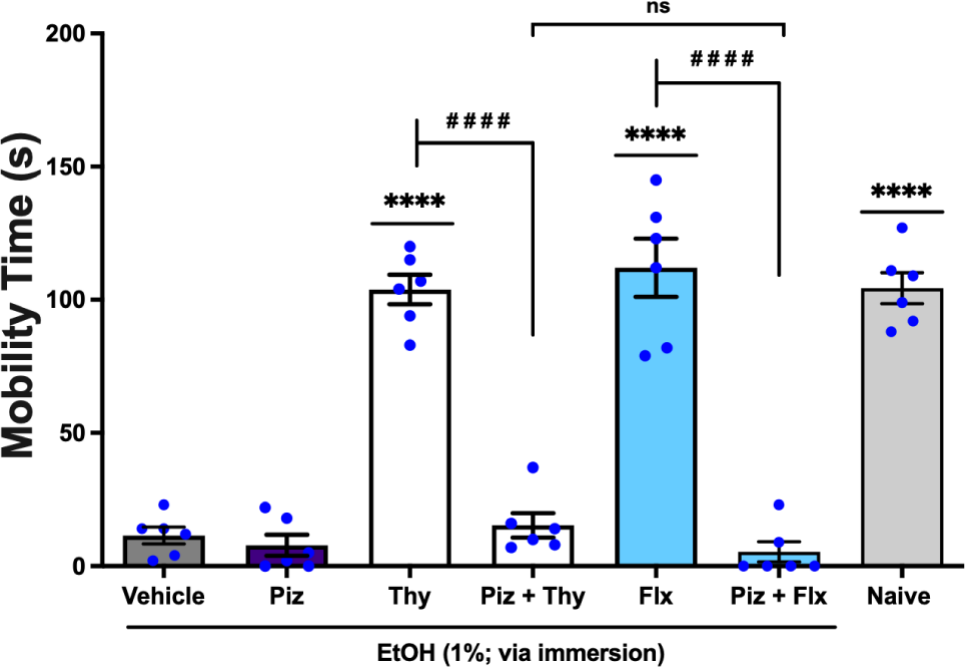
Effect of pizotifen (Piz; 5-HT_1_ and 5-HT_2*A*/2C_ antagonist; 0.8 mg/mL; 20 μL; *p.o.*) on the antidepressant-like activity of thymol (Thy, 0.01 mg/mL; 20 μL; *p.o.*) in the Zebrafish Tail Immobilization Test (ZTI), 0–5 min. Naive—untreated animals. Flx: fluoxetine, employed as the reference antidepressant (5.0 mg/mL; 20 μL; *p.o.*). Vehicle: 3% DMSO, orally administered in a volume of 20 μL. Data are presented as mean ± standard error of the mean (S.E.M.) for six animals per group. Statistical analysis was conducted using ANOVA followed by Tukey’s post hoc test (****p < 0.0001 *vs.* Vehicle; ^**# # # #**^ *p* < 0.0001 *vs.* thymol or Flx; ns – not significant = *p* > 0.05).

#### 2.3.3 Neuromodulation via the 5-HT_3*A*/3B_

The antidepressant-like activity of EOLg was observed following oral administration at 0.01 mg/mL in 20 μL was significantly reversed (*q* = 7.656, *p* < 0.001; EOLg *vs.* Gstn + EOLg) by granisetron (Gstn) at 0.5 mg/mL, orally administered in 20 μL an antagonist of the serotonergic system 5-HT_3*A*/3B_.^13^ This effect was significantly (*q* = 2.178, *p* < 0.05; Gstn + EOLg *vs.* Gstn + Flx) similar to the effect of Fluoxetine (Flx), used as a standard antidepressant, administered per os at 5.0 mg/mL in a volume of 20 μL, which also had a significantly reversed effect (*q* = 12.63, *p* < 0.0001; Flx *vs.* Gstn + Flx) by granisetron, Figure 7 (F _6, 35_ = 35.49). Thus, the reversal of the antidepressant-like effect of EOLg by pretreatment with granisetron suggests that its effects depend on the serotonergic 5-HT_3*A*/3B_ receptor.

**Figure 7:**
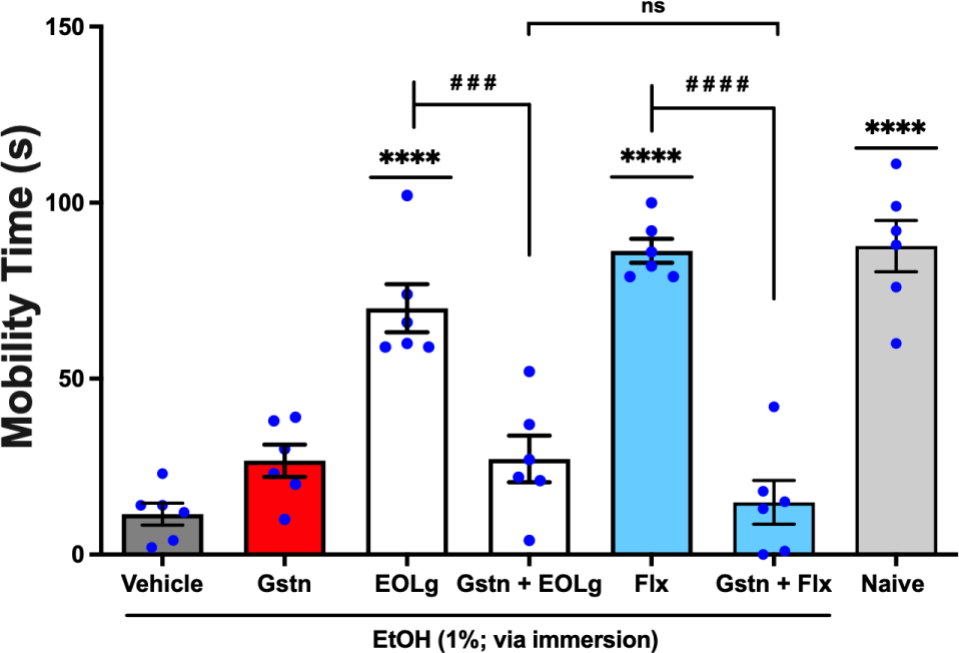
Effect of granisetron (Gstn; 5-HT_3*A*/3B_ antagonist; 0.5 mg/mL; 20 μL; *p.o.*) on the antidepressant-like effect of essential oil from *Lippia grata* leaves (EOLg; 0.01 mg/mL; 20 μL; *p.o.*) in the Zebrafish Tail Immobilization Test (ZTI), 0–5 min. Naive—untreated animals. Flx: fluoxetine, employed as the reference antidepressant (5.0 mg/mL; 20 μL; *p.o.*). Vehicle: 3% DMSO, orally administered in a volume of 20 μL. Data are presented as mean ± standard error of the mean (S.E.M.) for six animals per group. Statistical analysis was conducted using ANOVA followed by Tukey’s post hoc test (****p < 0.0001 *vs.* Vehicle; ^**# # #**^ *p* < 0.001 *vs.* EOLg; ^**# # # #**^ *p* < 0.0001 *vs.* Flx; ns—not significant = *p* > 0.05).

The antidepressant-like effect of activity of thymol was observed following per os administration at 0.01 mg/mL in 20 μL was also significantly reversed (*q* = 11.79, *p* < 0.0001; Thy *vs.* Gstn + Thy) by granisetron (Gstn) at 0.5 mg/mL, orally administered in 20 μL an antagonist of the serotonergic system 5-HT_3*A*/3B_.^13^ This effect was significantly (*q* = 1.299, *p* < 0.05; Gstn + Thy *vs.* Gstn + Flx) similar to the effect of Fluoxetine (Flx), used as a standard antidepressant, administered per os at 5.0 mg/mL in a volume of 20 μL, which also had a significantly reversed effect (*q* = 14.29, *p* < 0.0001; Flx *vs.* Gstn + Flx) by granisetron, Figure 8 (F _6, 35_ = 48.07). Thus, the reversal of the antidepressant-like effect of thymol by pretreatment with granisetron suggests that its effects depend on the serotonergic 5-HT_3*A*/3B_ receptor.

**Figure 8:**
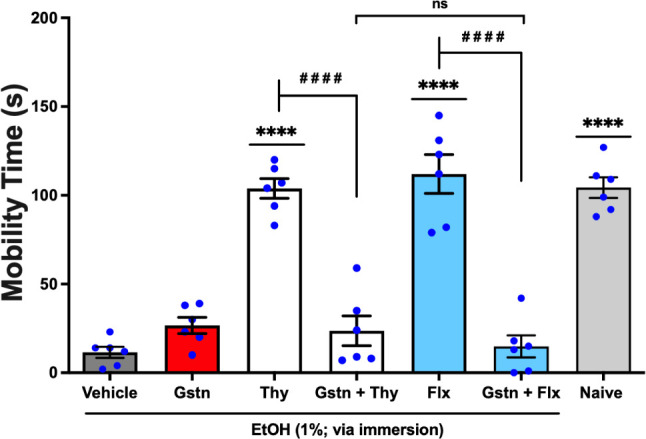
Effect of granisetron (Gstn; 5-HT_3*A*/3B_ antagonist; 0.5 mg/mL; 20 μL; *p.o.*) on the anxiolytic-like activity of thymol (Thy, 0.01 mg/mL; 20 μL; *p.o.*) in the Zebrafish Tail Immobilization Test (ZTI), 0–5 min. Naive—untreated animals. Flx: fluoxetine, employed as the reference antidepressant (5.0 mg/mL; 20 μL; *p.o.*). Vehicle: 3% DMSO, orally administered in a volume of 20 μL. Data are presented as mean ± standard error of the mean (S.E.M.) for six animals per group. Statistical analysis was conducted using ANOVA followed by Tukey’s post hoc test (****p < 0.0001 *vs.* Vehicle; ^**# # # #**^ *p* < 0.0001 *vs.* EOLg or Flx; ns—not significant = *p* > 0.05).

### 2.4 Molecular Docking Simulations

#### 2.4.1 Molecular Docking of Thymol with 5-HT_1B_ Receptor

To confirm the role of the serotonergic system in thymol’s effects, molecular docking simulations were conducted targeting various 5-HT receptors. Regarding the 5-HT_1B_ receptor, the most energetic cluster presented an energy loss of −274.10 kcal after complexation of the ligand, which suggests a relevant interaction when compared to the binding energy of the classical agonist fluoxetine (−313.84 kcal). In this receptor, thymol showed affinity and specificity to a binding site close to the specific site of fluoxetine, a ligand that modulates the binding sites of the 5-HT_1B_ receptor,^14^ involving residues that constitute secondary structures similar (α helix), Figure 9.

**Figure 9:**
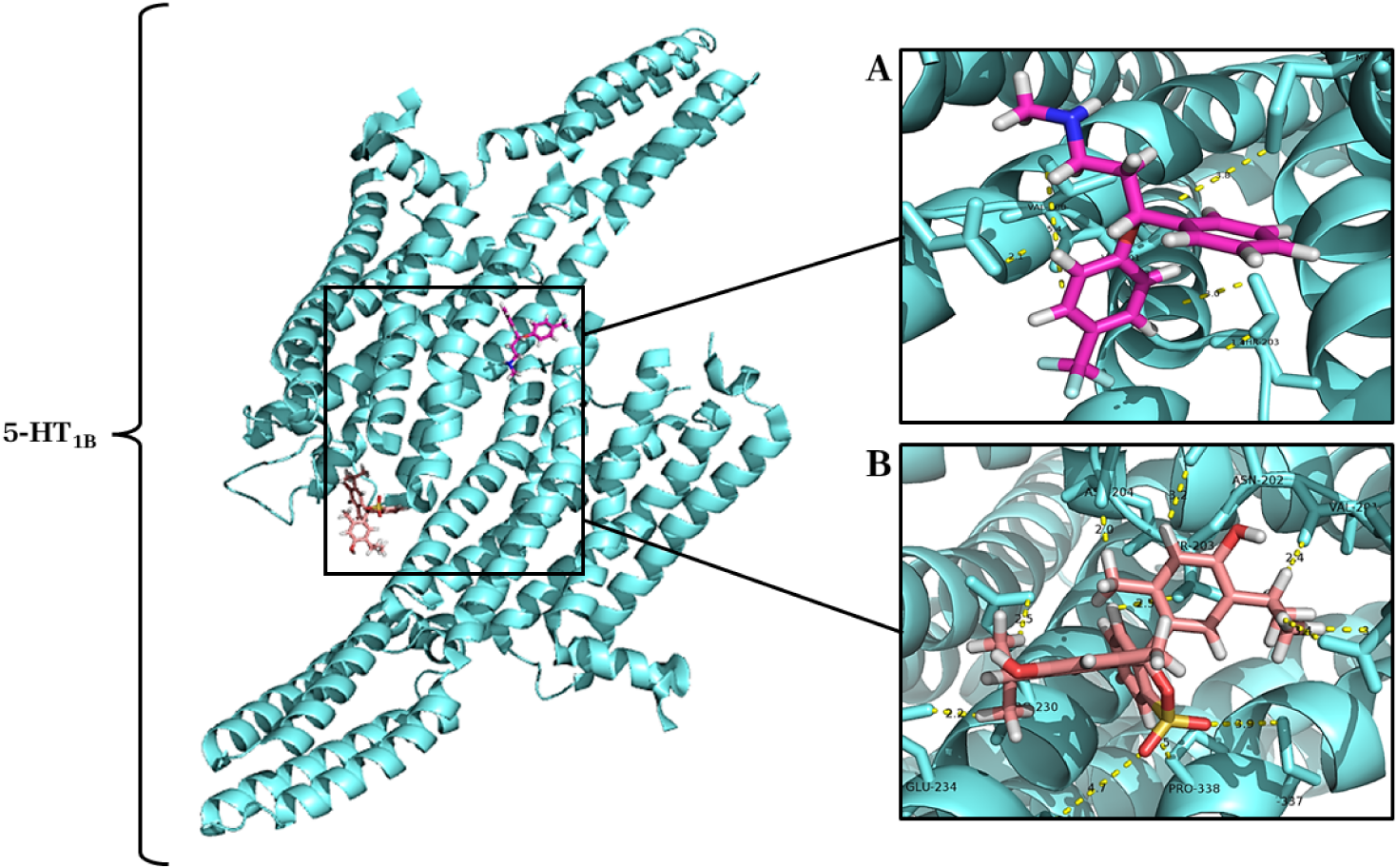
Complex formed between the 5-HT_1B_ receptor (PDB ID: 5 V54) (cyan cartoon) and the ligands thymol (salmon sticks) and fluoxetine (pink rose sticks): (A) Binding site of fluoxetine (pink rose) with the receptor 5-HT_1B_. (B) Thymol (salmon) binding site with the 5-HT_1B_ receptor.

During the complexation of fluoxetine (pink), used as a positive control, with the 5-HT_1B_ receptor, six chemical bonds ranging from 1.4 to 5.4 Å were identified, involving the interaction with five amino acid residues of the receptor: Thr203, Val201, Val200, Glu198 and Met337 (Figure 9A). The interaction between thymol and the 5-HT_1B_ receptor promoted 11 chemical bonds (2.0 to 4.7 Å), with recruitment of 11 amino acid residues: Glu198, Val200, Val201, Asn202, Asp204, Thr203, Arg230, Glu234, Val233, Pro338, and Met337 (Figure 9B).

#### 2.4.2 Molecular Docking of Thymol with the 5-HT_2A_ Receptor

Regarding the 5-HT_2A_ receptor, the most energetic cluster presented an energy loss of −169.52 kcal after complexation of the ligand, which suggests a similar interaction when compared to the binding energy of the classical agonist fluoxetine (−181.19 kcal). At this receptor, thymol exhibited affinity and specificity for a binding site distinct from that of fluoxetine, a direct-acting ligand targeting another 5-HT_2A_ receptor subtype. This finding suggests the presence of both primary and secondary binding sites within the receptor^15^ Figure 10.

**Figure 10:**
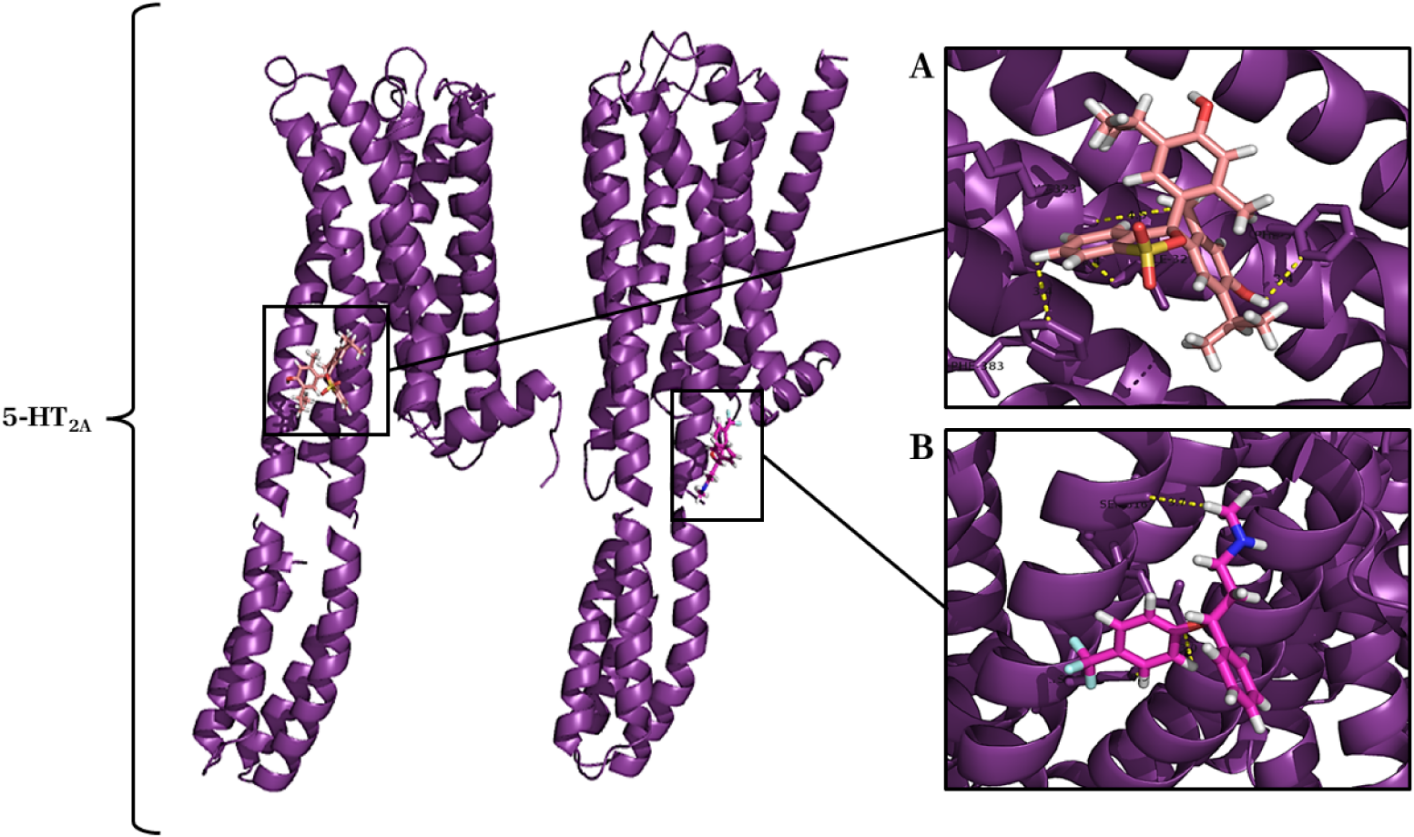
Complex formed between the 5-HT_2A_ receptor (PDB ID: 6A94) (purple cartoon) and the ligands thymol (salmon sticks) and fluoxetine (pink rose sticks): (A) Thymol binding site. (B) Fluoxetine binding site (pink).

The interaction between thymol and the 5-HT_2A_ receptor promoted 4 chemical bonds (3.4 to 4.5 Å) with the recruitment of 4 amino acid residues of the receptor: Ile327, Lys323, Phe383 and Phe330 (Figure 10A). In the complexation of fluoxetine (pink), positive control, with the 5-HT_2A_ receptor, 3 chemical bonds (0.9 to 3.7 Å) were observed with the recruitment of 3 amino acid residues of the receptor: Ser316, Gln319 and Lys323 (Figure 10B).

#### 2.4.3 Molecular Docking of Thymol with 5-HT_2C_ Receptor

The complex formed from the interaction between thymol and the 5-HT2C receptor showed that the most energetic cluster presented an energy loss of −307.08 kcal after the ligand was complexed, which suggests a strong and greater interaction compared to the binding energy of the classical agonist fluoxetine (−266.89 kcal). Thymol showed affinity and specificity for a binding site close to and similar to that of fluoxetine (direct-acting ligand of another 5-HT2 receptor subtype),^15^ Figure 11, making several chemical bonds and recruiting many amino acid residues.

**Figure 11:**
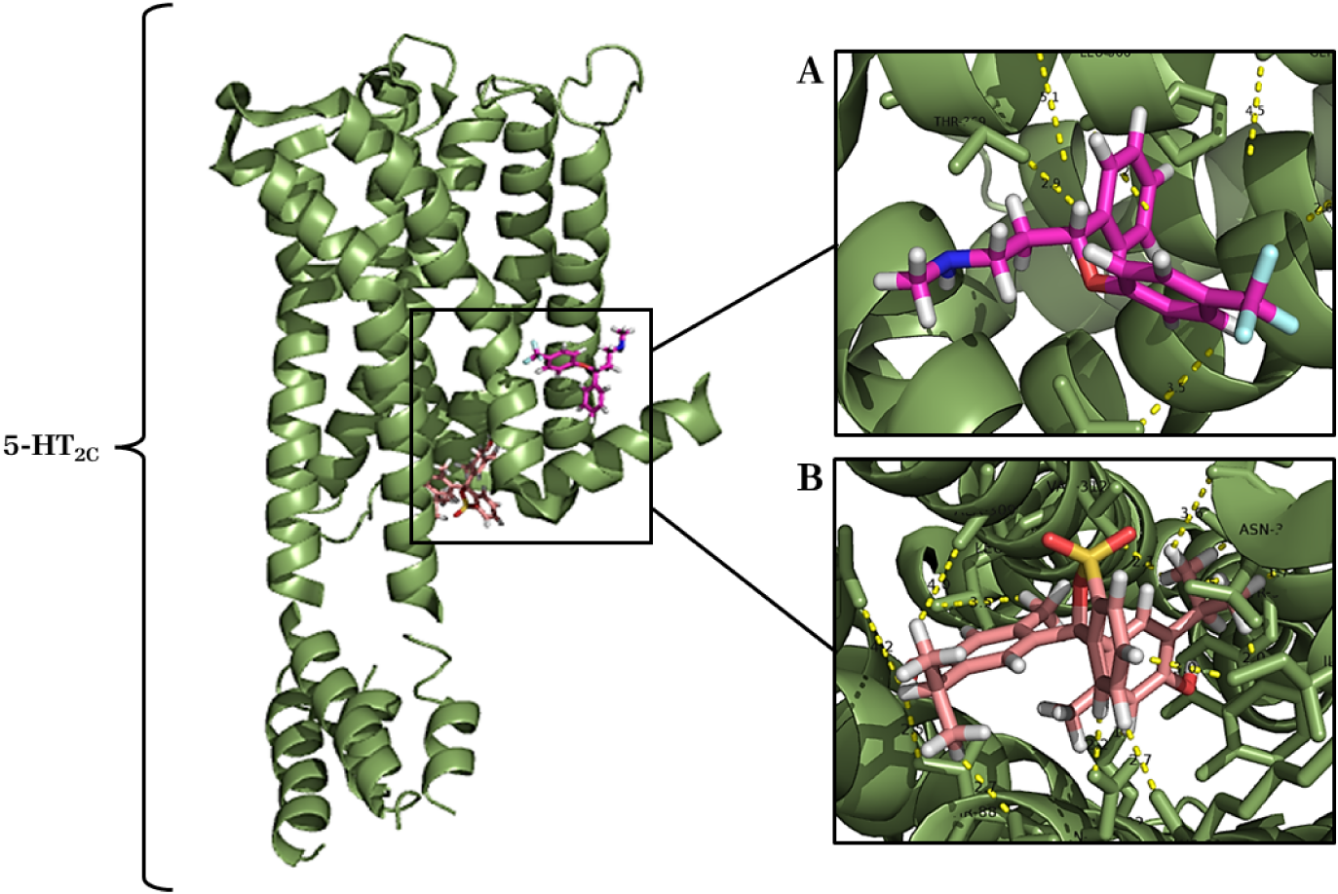
Complex formed between the 5-HT_2C_ receptor (PDB ID: 6BQH) (green cartoon) and the ligands thymol (salmon sticks) and fluoxetine (pink rose sticks): (A) Binding site of fluoxetine (pink rose) with the receptor 5-HT_2C_. (B) Thymol (salmon) binding site with the 5-HT_2C_ receptor.

In the complexation of fluoxetine (pink), positive control, with the 5-HT_2C_ receptor, 6 chemical bonds (2.6 to 5.1 Å) were observed with the recruitment of 6 amino acid residues of the receptor (Thr369, Leu366, Pro365, Gly362, Met66 and Leu383), Figure 11A. The interaction between thymol and the 5-HT_2C_ receptor promoted 15 chemical bonds (1.7 to 4.2 Å), with recruitment of 15 amino acid residues Asn86, Lys83, Ile374, Tyr375, Asn372, Phe371, Tyr368, Val367, Leu92, Asn89, Thr88, Arg152, Leu313, Val312 and Ala309), Figure 11B.

#### 2.4.4 Molecular Docking of Thymol with 5-HT_3A_ Receptor

The complex formed from the interaction between thymol and the 5-HT3A receptor showed that the most energetic cluster presented an energy loss of −195.98 kcal after the ligand’s complexation, which suggests a relevant interaction compared to the energy binding of the classical agonist fluoxetine (−240.95 kcal). Thymol showed affinity and specificity for a binding site different from fluoxetine, a ligand that interacts with 5-HT_3A_ receptor binding sites,^16^ making multiple chemical bonds and recruiting many amino acid residues (Figure 12).

**Figure 12:**
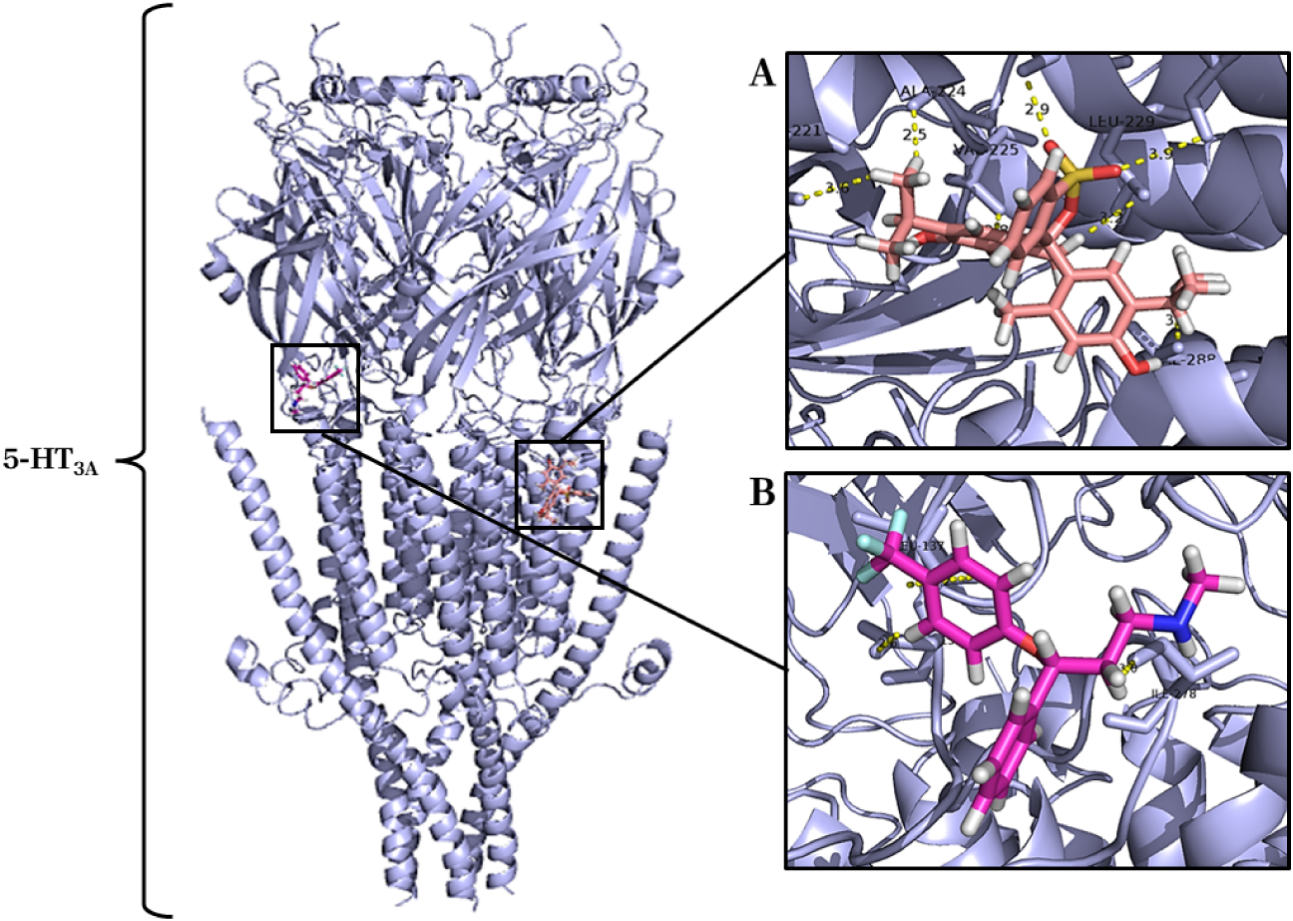
Complex formed between the 5-HT_3A_ receptor (PDB ID: 6W1Y) (light blue cartoon) and the ligand thymol (salmon sticks) and fluoxetine (pink pink sticks): (A) Binding site of thymol (salmon sticks) with the receptor 5-HT_3A_. (B) Fluoxetine binding site (pink rose) with the 5-HT_3A_ receptor.

The interaction between thymol and the 5-HT_3A_ receptor promoted 7 chemical bonds (2.5 to 3.9 Å) with the recruitment of 7 amino acid residues of the receptor ((Leu221, Ala224, Val225, Leu228, Leu229, Ile232 and Val288), Figure 12A. In the complexation of fluoxetine (pink), positive control, with the 5-HT_3A_ receptor, 3 chemical bonds (2.0 to 5.2 Å) were observed with the recruitment of 3 amino acid residues of the receptor (Asp138, Leu137 and Ile278), Figure 12B.

## 3 Discussion

In this study, adult zebrafish (*Danio rerio*) were employed as an alternative to rodents for standardizing a depression test, demonstrating that this model offers a cost-effective and highly reproducible approach. Consequently, we employed this method to evaluate the antidepressant potential of *Lippia grata* essential oil and the phytoceutical thymol. In addition, we investigated their *in vitro* antioxidant and anticholinesterase (AChE) potential.

*Lippia grata* Schauer is a shrub native to the areas of Northeastern Brazil and is commonly employed in traditional medicine to alleviate pain and inflammation. Despite its widespread use, scientific studies on this plant remain limited, especially concerning its pharmacological properties^17^ Studies indicate that the plant’s essential oil exhibits several therapeutic activities, including antioxidant, antineoplastic, anxiolytic, analgesic, and antimicrobial properties,^18^ primarily attributed to carvacrol and thymol in its composition.^19^

The chemical composition of this essential oil was previously characterized by Felix et al.^20^ using gas chromatography–mass spectrometry (GC-MS). Identification of constituents was based on the Kovats retention index (RI) and comparison of mass spectra with existing literature data. Essential oil yields were measured during different seasons: rainy (REO) at 1.7%, flowering (FEO) at 2.72%, and dry (DEO) at 1.2%. The analysis identified multiple compounds, including α-pinene, sabinene, β-pinene, myrcene, α-terpinene, p-cymene, limonene, carvacrol, thymol methyl ether, and thymol acetate. Thymol and 1,8-cineole emerged as the predominant constituents throughout all seasons, with concentrations of 58.46% and 9.43% in REO, 65.82% and 7.00% in FEO, and 73.49% and 13.58% in DEO, respectively. Together, these oxygenated monoterpenes represented 67.89% (REO), 72.82% (FEO), and 87.07% (DEO) of the total essential oil content, with the highest abundance noted in the dry season.

Toxicity assessments continue to rely heavily on animal models, particularly rodents, in preclinical laboratory evaluations of substances. Nevertheless, the use of mammals, along with the considerable number of animals required, raises ethical and practical issues, in addition to being costly and labor-intensive.^21^ In vivo studies were performed using adult zebrafish, which have emerged as a valuable complementary model to rodents in fields such as genetics, developmental biology, neurobiology, and toxicology.^22^ This species offers multiple advantages, including low maintenance costs, high adaptability to different environments, short reproductive cycles, prolific breeding capacity, and transparent embryos.^23^

Their small size in adulthood necessitates a reduction in the amounts of substances to be tested and dosed, as well as the quantities of reagents and materials used to treat and maintain the animals.^24^ The choice of zebrafish for neurological tests is justified by their rapid response to treatments, which facilitates observing effects over a relatively short period.^25^ The presence of organs and metabolic pathways in zebrafish that are analogous to those in humans allows for toxicological and biocompatibility assessments.^26^ Their rapid development, compared to mammals, significantly reduces the time required for experiments.^27^ The results of the oral administration of EOLg and thymol, presented in Table 1, confirm that the tested samples did not show toxicity. Furthermore, in the Open Field Test, conducted to assess possible locomotor changes in the animals (Figure 1), it was observed that both compounds affect the central nervous system (CNS) without compromising motor skills.

As an alternative model, implementing models such as zebrafish has gained significant attention. Zebrafish has demonstrated extensive results that are highly comparable to human conditions, making it a promising option. Due to its numerous advantages, this model has established itself as an ideal alternative for several scientific studies.^28^

In the antioxidant assays (Table 2), all samples demonstrated the ability to inhibit radicals. This potential for inhibiting oxidative radicals was assessed by measuring the inhibition of the DPPH and ABTS radicals, widely recognized methods for evaluating a substance’s ability to neutralize free radicals.^29^ Additionally, it is important to highlight that IC_50_ values below 50 μg.mL^–1^ are considered indicative of high antioxidant activity.^30^ Therefore, these findings reinforce the promising potential of the samples to function as natural antioxidants.

The antioxidant system, a collective of endogenously synthesized and exogenously derived antioxidants, is a reassuring testament to the body’s natural defense mechanisms. This system, which includes enzymes, small molecules, minerals, carotenoids, phenols, vitamins, and flavonoids, works in harmony to reduce oxidative stress.^31^

Medicinal plants are recognized sources of antioxidants and are widely acknowledged for their various health benefits, including potential protection against viral hepatic diseases and neurodegenerative disorders. This protective effect is associated with the role of antioxidants in reducing oxidative stress, a key factor in the progression of these conditions.^32^

In the realm of plant activities, the inhibition of AChE stands out as a significant strategy in the battle against neurodegenerative diseases. This promising approach reduces the degradation of acetylcholine, thereby preserving its function in the nervous system.^33^ The potential of phytochemicals found in plant extracts to combat neurodegenerative disorders such as Alzheimer’s disease and dementia is a testament to the power of nature. In addition to AChE inhibition, these compounds possess antioxidant, anti-inflammatory, and antiamyloid properties, which may contribute to neuroprotection and the reduction of oxidative stress, a key factor in the progression of these diseases.^34^

In addition to the antioxidant assays, another *in vitro* test performed was the AChE activity of EOLg and thymol (Table 2). Both demonstrated high enzyme inhibition, with particular emphasis on the essential oil. AChE inhibitors have been associated with hepatoprotective properties.^35^ Evidence suggests that molecules with anticholinesterase activity hold great potential for developing new drugs to treat neurodegenerative diseases, such as Alzheimer’s disease (AD). Currently, anticholinesterase drugs are the mainstay of pharmacotherapy for this condition. However, the number of drugs available for the treatment of AD remains limited, highlighting the need for further advances in this area.^36^ In this context, the design, synthesis, and modification of neuroprotective agents remain a central focus of research for developing of new drugs.^37^

Ethanol (EtOH) is a psychoactive substance that depresses the central nervous system (CNS) and is commonly used to identify effective drugs for combating depression in alternative models such as adult zebrafish.^38^ Thus, we used EtOH as a CNS depressant in aZF and evaluated the antidepressant-like effect of EOLg and the phytochemical thymol through the ZTI in aZF,^39^ using fluoxetine as an antidepressant control (Figure 2A). A recent study by de Araújo et al.^40^ investigated the neuropharmacological properties of *Mimosa tenuiflora* using adult zebrafish. The researchers employed an adapted depression model based on immobilization stress through calta in aZF, also investigating neuromodulation via the serotonergic system. In this protocol, a 1% ethanol solution was employed to induce depressive-like behavior, and locomotor activity was analyzed using ToxTrac software (version 2.98). Zebrafish movements were recorded along the axial plane for a duration of 5 min using a video camera. In our study, we adapted the same test but manually recorded the parameters, with calibrated and blinded observers, who were unaware of which groups had been applied. Nonetheless, it is noteworthy that both methods yielded comparable results, with no statistically significant differences observed. Thus, natural antioxidant products represent an effective alternative to mitigate the damage caused by ethanol metabolism, which generates reactive oxygen species (ROS) and contributes to the progression of various diseases.

It was demonstrated that EOLg and the phytochemical thymol effectively reduced depressive behavior induced by 1% ethanol (Figure 2B,C). The essential oil from *Lippia grata* leaves (EOLg) exhibited an effect similar to the antidepressant fluoxetine in adult zebrafish, significantly increasing the mobility time in the immobility test compared to the control group treated with the vehicle. Additionally, thymol’s effect was similar to that produced by fluoxetine, used as a positive control, indicating its antidepressant potential.

Research focusing on brain activity and pharmacological interventions has greatly advanced the understanding of the mechanisms underlying mental disorders, especially those related to depression and anxiety.^41^

In the mechanisms of neuromodulation via the 5-HT_2A_ serotonergic system, To explore the mechanism involved in the anxiolytic-like effects of EOLg and thymol, cyproheptadine a 5-HT_2A_ receptor antagonist was administered as a pretreatment. This intervention attenuated the observed anxiolytic-like responses suggesting that its actions involve 5-HT_2A_ receptors (Figures 3 and 4). In zebrafish, 5-HT levels are directly associated with movement disorders, anxiety, and depression. A decrease in this neurotransmitter has been linked to the emergence of anxiogenic behaviors.^42^

In this investigation, a single-dose pretreatment with pizotifen was administered (Figures 5 and 6) (5-HT_1_ and 5-HT_2*A*/2C_ receptor antagonist)^43^ and granisetron (Figures 7 and 8) (5-HT_3A/3B_ receptor antagonist),^44^ as well as EOLg and thymol, reversed anxiogenic behavior. These results suggest a possible serotonergic system activation via 5-HT_1_ and 5-HT_2*A*/2C_ receptors.

Depression is a multifactorial disorder involving a range of mechanisms, such as the interplay between various neurotransmitters, biochemical pathways, neural circuits, specific brain regions, and the interaction between the immune and nervous systems.^45^ Among the various systems involved, the serotonergic pathway exerts a multifaceted influence on both anxiety and depression. This emphasizes the need to investigate alterations in the expression of 5-HT receptors related to these behavioral states.^46^ Serotonin (5-hydroxytryptamine, 5-HT) receptors comprise at least 15 distinct subtypes, several of which have been linked to the development of depressive symptoms.^47^

Serotonin (5-hydroxytryptamine, 5-HT) is an essential neurotransmitter that regulates numerous physiological processes, exerting its effects both within the central nervous system and throughout peripheral tissues.^48^ Its effects are mediated by activating a family of receptors divided into seven subtypes. With the exception of the 5-HT3 receptor, which functions as a ligand-gated ion channel, all other serotonin (5-HT) receptors belong to the family of G-protein coupled receptors (GPCRs). These receptors regulate multiple signal transduction processes^49^ and are involved in several pathologies, such as depression and anxiety.^50^

Fluoxetine is a widely used medication for the treatment of anxiety and depression and is the main representative of the selective serotonin reuptake inhibitor (SSRI) class. Previous studies suggest that its therapeutic effects, when administered orally, may be mediated, at least in part, by its action on the brain via the vagus nerve.^51^

Molecular docking simulations demonstrated that thymol interacts significantly with various serotonergic (5-HT) receptors, reinforcing its potential to modulate the serotonergic system (Figures 9–12). These interactions were analyzed for affinity, energy loss, and recruitment of amino acid residues, highlighting the specificity of thymol for each receptor subtype. Regarding the 5-HT_1B_ receptor (Figure 9), thymol showed a notable interaction, with considerable energy loss and affinity for a binding site near that occupied by fluoxetine. The complexation involved 11 amino acid residues and established 11 chemical bonds, suggesting an interaction mechanism comparable to the classical agonist.

At the 5-HT_2A_ receptor, thymol showed affinity for a different binding site than fluoxetine, indicating the possibility of both primary and secondary binding sites at this receptor. The interaction resulted in four chemical bonds involving four amino acid residues, suggesting a specific profile for this subtype (Figure 10).

The 5-HT_2C_ receptor exhibited the most prominent interaction with thymol (Figure 11), displaying an affinity for a binding site near fluoxetine. Thymol established 15 chemical bonds involving 15 amino acid residues, suggesting a stronger and more significant interaction than the classical agonist. Finally, at the 5-HT_3A_ receptor, thymol interacted differently, occupying a distinct binding site from fluoxetine. Seven chemical bonds with seven amino acid residues were observed, indicating another relevant and specific interaction for this receptor subtype (Figure 12).

Recent developments in computational techniques have introduced a powerful array of tools, including molecular docking, molecular dynamics (MD) simulations, quantitative structure–activity relationship (QSAR) modeling, and pharmacokinetic profiling. These methodologies enable researchers to analyze extensive data sets, identify potential drug candidates, optimize their characteristics, and expedite the drug discovery process.^52^ Beyond surpassing traditional screening methods, they also provide detailed insights into the mechanisms underlying therapeutic compound actions.^53^

## 4 Conclusion

Our findings desmonstrate that the samples are non-toxic to adult zebrafish (*D. rerio*). Flx presented an antidepressant effect, but with a sedative effect while EOLg and thymol exhibited an antidepressant effect without a sedative effect and via serotonergic systems. *In silico* tests demonstrated the affinity of the ligands for the 5-HT_1B_, 5-HT_2A_, 5-HT_2C_, and 5-HT_3A_ receptors, with favorable binding energies, reflecting interactions in regions of high flexibility of the receptors. *In vitro* tests suggested the antioxidant and neuroprotective potential of EOLg and thymol against the acetylcholinesterase (AChE) enzyme for the tested samples. This work reinforces the relevance of plant-derived natural products in treating neurological diseases.

## 5 Material and Methods

### 5.1 Drugs and Chemical Reagents

The compounds utilized in this study included Diazepam (Dzp, Neo Química), Fluoxetine (Flx, EMS), Cyproheptadine (Cypro), Pizotifen (Piz), Granisetron (Gstn), 2,2-diphenyl-1-picrylhydrazyl (DPPH), quercetin, gallic acid, and physostigmine, all of which were obtained from Sigma-Aldrich Corp., USA.

### 5.2 Test Samples

The essential oil obtained by Felix et al.^20^ from the *Lippia grata* leaves (EOLg), collected in the Serra do Gadelha region (6° 26′19”S; 39° 15′ 53” W), Iguatu-CE, Brazil, and kindly donated by Teacher Dra. Selene Maia de Morais from the Department of Chemistry, Chemistry Course, Center of Sciences and Technology, State University of Ceará, was used. Thymol (Sigma-Aldrich, USA), the major constituent of EOLg, was also used. Both samples were stored in a refrigerator (5 °C) in our laboratory until use.

### 5.3 Antioxidant Activity

The antioxidant capacity was assessed through DPPH (2,2-diphenyl-1-picrylhydrazyl) and ABTS [2,2’-azino-bis­(3-ethylbenzothiazoline-6-sulfonic acid)] assays, following the protocols described by Becker et al.^54^ and Re et al.,^55^ respectively, with modifications. Both assays were conducted in 96-well flat-bottom microplates, and absorbance readings were obtained using a BioTek ELISA reader (model ELX 800). Test samples and positive controls were prepared from a 2.0 mg·mL^–1^ stock solution and diluted to final concentrations of 100, 50, 25, 12.5, 6.25, and 3.12 μg·mL^–1^. Absorbance was measured at 490 nm for the DPPH^•^ radical after 60 min of incubation and at 630 nm for the ABTS^+•^ radical after 10 min. Solutions containing all reagents, except the sample, were used as negative controls. Data were adjusted to eliminate interference from the natural colors of the extracts. Quercetin and gallic acid were used as comparison antioxidant standards.

### 5.4 Antiacetylcholinesterase Activity

The inhibitory effect on acetylcholinesterase (AChE) activity was measured in 96-well flat-bottom plates using a BioTek ELISA reader (model ELX 800) with “Gen5 V2.04.11” software, following the method described by Ellman et al.^56^ Each well contained 25 μL of acetylthiocholine iodide (15 mM), 125 μL of 5,5́-dithiobis­(2-nitrobenzoic acid) (DTNB) in Tris/HCl buffer (50 mM, pH 8) with 0.1 M NaCl, 0.02 M MgCl_2_·6H_2_O, and 3 mM DTNB, 50 μL of Tris/HCl buffer (50 mM, pH 8) containing 0.1% bovine serum albumin (BSA), and 25 μL of the extract diluted 10-fold in Tris/HCl (50 mM, pH 8) to reach a final concentration of 2.0 mg·mL^–1^.^57^ Sample and positive control dilutions were prepared from a 2.0 mg·mL^–1^ stock to final concentrations of 100, 50, 25, 12.5, 6.25, and 3.12 μg·mL^–1^. Absorbance readings at 405 nm were taken for 30 s initially, followed by the addition of 25 μL of acetylcholinesterase enzyme (0.25 μ/mL). Absorbance was then recorded every minute over 25 min of incubation. Negative controls contained all reagents except the test sample. Data were corrected to account for any extract color interference. Galantamine and physostigmine served as positive controls.

### 5.5 Adult Zebrafish (*Danio rerio*)

Adult zebrafish (*Danio rerio*) of both sexes, approximately 90 days old, with an average length of 3.5 ± 0.5 cm and weight of 0.4 ± 0.1 g, exhibiting short tails, were sourced from Agroquímica: Comércio de Produtos Veterinários LTDA, located in Fortaleza, Ceará, Brazil. The animals were acclimatized for 24 h in glass tanks measuring 40 × 20 × 25 cm, filled with water treated with ProtecPlus antichlor and equipped with air pumps and submerged filtration systems. The tanks were maintained at 25 °C with a pH of 7.0, under a controlled photoperiod of 14 h light and 10 h dark. Prior to experimentation, fish were fed ad libitum with Spirulina 24 h in advance. All procedures adhered to ethical guidelines and received approval from the Animal Use Ethics Committee of the State University of Ceará (CEUA-UECE), protocol number 04009489/2023.

### 5.6 General Protocol

The zebrafish assays were performed following the protocols described by Magalhães et al.^58,59^ On the day of the experiment, fish were randomly chosen, gently transferred onto a damp sponge, and administered test or control samples per os (*p.o.*).^60^ Following treatment, each animal was individually placed in a 250 mL beaker containing 150 mL of aquarium water for acclimation. Oral administration was carried out using a 20 μL automatic pipet fitted with sterile tips. Behavioral observations were conducted by calibrated observers blinded to the treatment groups.^58,59^

#### 5.6.1 Nonclinical Safety Assessment

##### 5.6.1.1 Acute Toxicity 96 h

The acute toxicity assay was conducted in accordance with OECD guidelines^61^ and following the protocol established by Batista et al.^62^ Adult zebrafish (aZF, *n* = 6 per group) received treatments per os with 20 μL of EOLg or thymol at concentrations of 0.01 or 0.1 or 1.0 mg/mL; fluoxetine (Flx) at 5.0 or 10 or 15 mg/mL; or vehicle control (3% DMSO). Following administration, the fish were maintained at rest. Mortality was monitored every 24 h over a 96-h period to calculate the Lethal Concentration 50 (LC_50_), defined as the dose causing death in 50% of the subjects.

##### 5.6.1.2 Locomotor Activity (Open Field Test)

The Open Field Test^58^ was conducted to assess potential changes in motor coordination caused by sedation and/or muscle relaxation. Adult zebrafish (*n* = 6 per group) were treated per os (20 μL) with EOLg or thymol at concentrations of 0.01 or 0.1 or 1.0 mg/mL; fluoxetine (Flx) at 5.0 or 10 or 15 mg/mL; vehicle (3% DMSO); or diazepam (DZP; 10 mg/mL). A naïve group with no treatment and an untreated negative control group (*n* = 6) were also included. One hour postadministration, fish were individually placed in glass Petri dishes (100 × 15 mm) subdivided into horizontal quadrants and filled with aquarium water. Locomotor activity was evaluated by recording the number of line crossings (LC) over a 5 min period.

#### 5.6.2 Antidepressant-Like Effect

The antidepressant-like effect of the test samples was performed through the Zebrafish Tail Immobilization Test (ZTI), according to methodologies described by Kordjazy et al.,^63^ de Melo et al.,^38^ Demin et al.,^39^ with adaptations.

a) Experiment 1: the antidepressant effect of fluoxetine (Flx; control) was standardized. In this test, aZF (*n* = 6/group) were administered 20 μL per os of fluoxetine (Flx; 5.0 or 10 or 15 mg/mL) or vehicle (3% DMSO).
b) Experiment 2: the antidepressant effect of EOLg or thymol was evaluated, using the lowest effective dose of Flx as control (See results section). In this test, aZF (*n* = 6/group) administered 20 μL per os with 20 μL of LgEO or thymol (0.01 or 0.1 or 1.0 mg/mL) or Flx (Antidepressant control; 5.0 mg/mL). An untreated group was included (Naïve) in both experiments.

After 1 h of treatments, the animals were individually immersed in EtOH [(1%; Central Nervous System (CNS) depressant agent],^38^ for 30 min, with the exception of the naïve group. Subsequently, the aZF were individually subjected to the Tail Immobilization Test (TIC) and the antidepressant-like effect was characterized by the increase in mobility time (s) (MT) during 5.0 min of analysis.

##### 5.6.2.1 Neuromodulation via the Serotonergic System (5-HT)

The role of the serotonergic system in mediating the antidepressant-like effects of the lowest concentrations of EOLg or thymol was investigated using the Zebrafish Tail Immobilization Test (ZTI), following the protocols outlined by Demin et al.^39^ and employing 5-HT antagonists as described by Benneh et al.^13^ Initially, animals (*n* = 6 per group) received 20 μL treatments of:

Group A: EOLg or thymol (0.01 mg/mL administered per os);

Group B: fluoxetine (Flx; reference antidepressant (5.0 mg/mL; administered per os);

Group C: cyproheptadine (Cypro; 5-HT_2A_ antagonist; 0.8 mg/mL; administered per os);

Group D: pizotifen (Piz; 5-HT_1_ and 5-HT_2*A*/2C_ antagonist; 0.8 mg/mL; administered per os);

Group E: granisetron (Gstn; 5-HT_3*A*/3B_ antagonist; 0.5 mg/mL; administered per os);

Group F–H—Cyproheptadine (Cypro; 5-HT_2A_ antagonist; 0.8 mg/mL; administered per os), 15 min before treatment with 20 μL of EOLg or thymol (0.01 mg/mL; administered per os) or Fluoxetine (Flx; 5.0 mg/mL; administered per os);

Group I–K—Pizotifen (Piz; 5-HT_1_ and 5-HT_2*A*/2C_ antagonist; 0.8 mg/mL; administered per os), 15 min before treatment with 20 μL of EOLg or thymol (0.01 mg/mL; administered per os) or Fluoxetine (Flx; 5.0 mg/mL; administered per os);

Group L-N—Granisetron (Gstn; 5-HT_3*A*/3B_ antagonist; 0.5 mg/mL; administered per os), 15 min before treatment with 20 μL of EOLg or thymol (0.01 mg/mL; administered per os) or Fluoxetine (Flx; 5.0 mg/mL; administered per os);

Group O—vehicle (3% DMSO; 20 μL; administered per os);

Group P—animals without treatment (Naïve).

After 1 h of oral treatments, the animals were individually immersed in EtOH (1% central nervous system depressant) for 30 min, with the exception of the naive group.^38^ Subsequently, the animals were subjected to the Zebrafish Tail Immobilization Test (ZTI), and the antidepressant-like effect was characterized by an increase in mobility time (MT) during 5 min of analysis.

### 5.7 Molecular Docking

The interaction between serotonergic receptors (5-HT_1B_, 5-HT_2A_, 5-HT_2C_, 5-HT_3A_) and ligands (Thymol and the positive control Fluoxetine) was analyzed *in silico* using molecular docking simulations. The three-dimensional structures of the ligands Thymol and Fluoxetine were obtained from the PubChem database (CID: 65565 and 3386, respectively) and minimized. The three-dimensional structures of the 5-HT_1B_, 5-HT_2A_, 5-HT_2C_, and 5-HT_3A_ receptors were obtained from the Protein Data Bank, with the following PDB codes: 5 V54, 6A94, 6BQH, and 6W1Y, respectively. The ligands were removed from the 5-HT2A receptor structure before performing the molecular docking simulation. The structures were determined by X-ray crystallography, with resolutions ranging from 2.30 to 2.97 Å. The LigPrep protocol and the Epik tool^64^ were used to prepare the ligands. Molecular docking simulations were performed using HEX software, version 8.0.0 (Macindoe et al., 2010), which automatically adjusted the docking process based on the interaction energies between the ligands and all potential interaction sites on the surface of the serotonergic receptors. The resulting clusters were analyzed using PyMol, version 1.4.7,^65^ which allowed for a detailed investigation of the complexes.

### 5.8 Statistical Analysis

For in vitro experiments, all samples were evaluated in triplicate. In the in vivo studies, data are presented as mean ± standard error of the mean (SEM) for groups of six animals. After verifying data normality and homogeneity of variances, group comparisons were performed using one-way analysis of variance (ANOVA), followed by Tukey’s post hoc test. Statistical analyses were conducted with GraphPad Prism version 9.0. A p-value of less than 0.05 was considered statistically significant.
